# Supplementary material for: Disparities in Metabolic Conditions and Cancer Characteristics among Hispanic Women with Breast Cancer: A Multi-Institutional Study
Source: Cancers (Basel). 2022 Jul 14;14(14):3411. doi: 10.3390/cancers14143411 (PMC9317401; doi:10.3390/cancers14143411)
Supplement: Supplementary file 1 [file cancers-14-03411-s001.zip › cancers-1786441-supplementary.pdf]

Supplementary Table S1. Adjusted factors associated with Mexican Hispanics compared to Non-Mexican Hispanics

	OR*	95% CI		p-value
**Diabetes Mellitus**	3.45	2.02	5.88	<0.001
**Hypertension**	0.79	0.51	1.21	0.281
**Obesity**	1.76	1.19	2.60	0.005
**Dyslipidemia**	0.18	0.11	0.27	<0.001
**Postmenopausal**	1.84	1.23	2.77	0.003
**Diagnosis-IDC (reference)**				
Lobular	0.44	0.24	0.79	0.006
Lobular and Ductal	0.20	0.07	0.56	0.002
Other	0.89	0.42	1.86	0.751
**Stage-Early (reference)**				
Advanced	1.45	0.90	2.34	0.124
Unknown	0.83	0.53	1.31	0.43
**Surgery-None (reference)**				
Lumpectomy	5.47	2.94	10.20	<0.001
Mastectomy	2.39	1.34	4.27	0.003
Unknown	0.20	0.05	0.85	0.029

* **OR** Odds ratio, **CI** confidence interval; **IDC** invasive ductal carcinoma

Supplementary Table S2. Distribution of patient characteristics between Mexicans compared to non-Mexicans among premenopausal women

	Ethnicity		
	**Mexicans**	**Non-Mexicans**	**p-value**
**Demographics**			
**N**	280	65	
**Age (mean (SD))**	42.9 (5.1)	44.2 (4.9)	**0.057**
**BMI, (mean (SD))**	30.0 (6.0)	28.0 (5.7)	**0.018**
**Diagnoses**			**0.002**
Ductal	255 (91.1%)	49 (75.4%)	
Lobular	13 (4.6%)	6 (9.2%)	
Ductal and Lobular (mixed)	2 (0.7%)	3 (4.6%)	
Other	10 (3.6%)	7 (10.8%)	
**Tumor Characteristics**			
**Stage of tumor**			**0.11**
Stage I/II (early)	132 (47.1%)	30 (46.2%)	
Stage III/IV (adavnced)	88 (31.4%)	14 (21.5%)	
Unknown	60 (21.4%)	21 (32.3%)	
**Type of surgery**			**<0.001**
None	15 (5.4%)	8 (12.3%)	
Lumpectomy	142 (51.1%)	26 (40.0%)	
Mastectomy	121 (43.5%)	28 (43.1%)	
Unknown	0 (0.0%)	3 (4.6%)	
**Receptor subtype**			
Estrogen receptor	175 (63.9%)	57 (87.7%)	**<0.001**
Progesterone receptor	157 (57.5%)	50 (76.9%)	**0.004**
HER2 receptor	49 (20.4%)	13 (20.0%)	**0.94**
Hormonal receptor	180 (64.3%)	57 (87.7%)	**<0.001**
Triple-Negative Breast Cancer	65 (27.2%)	8 (12.3%)	**0.013**
**Comorbidities**			
Diabetes Mellitus	37 (13.2%)	8 (12.3%)	**0.84**
Hypertension	58 (20.7%)	16 (24.6%)	**0.49**
Dyslipidemia	35 (12.5%)	22 (33.8%)	**<0.001**
Obesity	125 (44.6%)	24 (36.9%)	**0.26**
Coronary artery disease	3 (1.1%)	0 (0.0%)	**0.40**
Metabolic abnormalities	17 (6.1%)	9 (13.8%)	**0.032**
Any comorbidities	168 (60.0%)	40 (61.5%)	**0.82**
**Number of comorbidities**			**0.20**
0	112 (40.0%)	25 (38.5%)	
1	107 (38.2%)	22 (33.8%)	
2	44 (15.7%)	9 (13.8%)	
3	8 (2.9%)	6 (9.2%)	
4	9 (3.2%)	3 (4.6%)	

SD standard devaition, **HER2** human epidermal receptor 2 neu

Supplementary Table S3. Distribution of patient characteristics by Hispanic origin.

	Mexican	Puerto Rican	Cuban	South/ Central American	Spanish NOS/ Hispanic NOS	Dominican Republic	p-value
**Demographics**							
N	851	5	11	21	129	3	
**Age (mean (SD))**	56.1 (11.9)	47.2 (5.5)	55.6 (15.3)	57.9 (10.0)	55.4 (11.8)	64.3 (11.2)	**0.40**
**BMI (mean (SD))**	30.9 (6.2)	34.0 (2.5)	27.3 (6.4)	26.4 (4.8)	28.1 (5.9)	25.3 (0.6)	**< 0.001**
**Premenopausal women**	280 (32.9%)	4 (80.0%)	4 (36.4%)	7 (33.3%)	50 (38.8%)	0 (0.0%)	**0.15**
**Tumor characteristics**							
**Diagnoses**							**< 0.001**
Ductal	734 (86.6%)	3 (60.0%)	9 (81.8%)	17 (81.0%)	93 (72.1%)	2 (66.7%)	
Lobular	53 (6.3%)	0 (0.0%)	2 (18.2%)	2 (9.5%)	18 (14.0%)	1 (33.3%)	
Ductal and Lobular	9 (1.1%)	0 (0.0%)	0 (0.0%)	2 (9.5%)	9 (7.0%)	0 (0.0%)	
Other	52 (6.1%)	2 (40.0%)	0 (0.0%)	0 (0.0%)	9 (7.0%)	0 (0.0%)	
**Stage of tumor**							**0.44**
Stage I/II	420 (49.4%)	2 (40.0%)	5 (45.5%)	7 (33.3%)	61 (47.3%)	2 (66.7%)	
Stage III/IV	236 (27.7%)	1 (20.0%)	2 (18.2%)	4 (19.0%)	35 (27.1%)	0 (0.0%)	
Unknown	195 (22.9%)	2 (40.0%)	4 (36.4%)	10 (47.6%)	33 (25.6%)	1 (33.3%)	
**Type of surgery**							**< 0.001**
None	62 (7.4%)	2 (40.0%)	2 (18.2%)	2 (9.5%)	24 (18.6%)	0 (0.0%)	
Lumpectomy	455 (54.0%)	2 (40.0%)	6 (54.5%)	8 (38.1%)	37 (28.7%)	1 (33.3%)	
Mastectomy	321 (38.1%)	1 (20.0%)	2 (18.2%)	11 (52.4%)	63 (48.8%)	2 (66.7%)	
Unknown	4 (0.5%)	0 (0.0%)	1 (9.1%)	0 (0.0%)	5 (3.9%)	0 (0.0%)	
**Receptor subtype**							
ER+	567 (68.6%)	5 (100.0%)	7 (63.6%)	18 (85.7%)	98 (76.0%)	1 (33.3%)	**0.084**
PR+	482 (58.4%)	4 (80.0%)	7 (63.6%)	15 (71.4%)	80 (62.0%)	1 (33.3%)	**0.58**
HER2+	129 (18.1%)	0 (0.0%)	1 (9.1%)	1 (4.8%)	28 (21.7%)	1 (33.3%)	**0.33**
HR+	578 (67.9%)	5 (100.0%)	7 (63.6%)	18 (85.7%)	98 (76.0%)	1 (33.3%)	**0.067**
TNBC	158 (22.2%)	0 (0.0%)	4 (36.4%)	3 (14.3%)	26 (20.2%)	1 (33.3%)	**0.55**
**Comorbidities**							
Diabetes Mellitus	237 (27.8%)	1 (20.0%)	2 (18.2%)	2 (9.5%)	19 (14.7%)	0 (0.0%)	**0.012**
Hypertension	322 (37.8%)	2 (40.0%)	5 (45.5%)	10 (47.6%)	58 (45.0%)	1 (33.3%)	**0.65**
Hyperlipidemia	212 (24.9%)	4 (80.0%)	6 (54.5%)	13 (61.9%)	60 (46.5%)	3 (100.0%)	**< 0.001**
Obesity	434 (51.0%)	5 (100.0%)	2 (18.2%)	5 (23.8%)	43 (33.3%)	0 (0.0%)	**< 0.001**
Coronary Artery Disease	36 (4.2%)	0 (0.0%)	1 (9.1%)	1 (4.8%)	5 (3.9%)	0 (0.0%)	**0.96**
Metabolic abnormalities	184 (21.6%)	2 (40.0%)	2 (18.2%)	4 (19.0%)	20 (15.5%)	0 (0.0%)	**0.47**
Any comorbidities	614 (72.2%)	5 (100.0%)	8 (72.7%)	16 (76.2%)	96 (74.4%)	3 (100.0%)	**0.63**
**Number of comorbidities**							**0.76**
0	237 (27.8%)	0 (0.0%)	3 (27.3%)	5 (23.8%)	33 (25.6%)	0 (0.0%)	
1	271 (31.8%)	1 (20.0%)	4 (36.4%)	7 (33.3%)	39 (30.2%)	2 (66.7%)	
2	159 (18.7%)	2 (40.0%)	2 (18.2%)	5 (23.8%)	37 (28.7%)	1 (33.3%)	
3	120 (14.1%)	1 (20.0%)	1 (9.1%)	3 (14.3%)	13 (10.1%)	0 (0.0%)	
4	64 (7.5%)	1 (20.0%)	1 (9.1%)	1 (4.8%)	7 (5.4%)	0 (0.0%)	

ER Estrogen Receptor, **PR** Progesterone receptor, **HR** Hormone Receptor, **TNBC** Triple-Negative Breast Cancer, **HER2** human epidermal receptor 2 neu, **SD** standard deviation, NOS Not Otherwise Specified

Supplementary Table S4. Association of Hispanic origin with breast cancer subtypes (ER, PR, HR, and Triple Negative) within premenopausal women.

	ER+*				PR+*			
	PR**	95% CI		p-value	PR**	95% CI		p-value
**Non-Mexican**	1.37	1.18	1.59	<0.001	1.38	1.14	1.67	0.001
**Diabetes Mellitus**	1.2	1	1.45	0.052	1.04	0.8	1.35	0.764
**Hypertension**	1.14	0.97	1.33	0.111	1.18	0.99	1.41	0.067
**Obesity**	1.05	0.9	1.21	0.542	0.99	0.84	1.18	0.938
**Dyslipidemia**	0.86	0.7	1.06	0.168	0.85	0.67	1.09	0.198
**Diagnosis-IDC (reference)**								
Lobular	1.37	1.14	1.65	0.001	1.47	1.19	1.81	<0.001
Lobular and Ductal	1.29	1.13	1.47	<0.001	1.4	1.16	1.69	<0.001
Other	0.89	0.59	1.35	0.594	0.57	0.28	1.18	0.129
**Stage-Early (reference)**								
Advanced stage	0.93	0.77	1.13	0.465	0.91	0.74	1.13	0.406
Stage Unknown	1.1	0.93	1.28	0.26	0.98	0.8	1.19	0.825
**Surgery-None (reference)**								
Lumpectomy	1.2	0.85	1.69	0.291	1.63	0.96	2.75	0.068
Mastectomy	1.15	0.82	1.6	0.429	1.55	0.92	2.61	0.097
Unknown	1.37	0.96	1.95	0.081	1.85	1.08	3.16	0.025
	HR+*				TNBC*			
**Non-Mexicans**	1.39	1.2	1.61	<0.001	0.43	0.21	0.87	0.019
**Diabetes Mellitus**	1.14	0.94	1.38	0.172	0.72	0.35	1.47	0.368
**Hypertension**	1.21	1.04	1.4	0.013	0.66	0.37	1.18	0.159
**Obesity**	1.02	0.89	1.18	0.742	1.25	0.82	1.91	0.303
**Dyslipidemia**	0.86	0.7	1.06	0.155	1.55	0.91	2.64	0.109
**Diagnosis-IDC (reference)**								
Lobular	1.37	1.15	1.64	<0.001	1.54	0.65	3.65	0.328
Lobular and Ductal	1.26	1.09	1.46	0.002				
Other	0.77	0.49	1.19	0.234				
**Stage-Early (reference)**								
Advanced	0.96	0.8	1.15	0.671	0.89	0.57	1.41	0.624
Unknown	1.04	0.89	1.22	0.618	1.03	0.61	1.73	0.913
**Surgery-None (reference)**								
Lumpectomy	1.2	0.85	1.7	0.292	0.45	0.23	0.89	0.021
Mastectomy	1.11	0.79	1.56	0.534	0.55	0.29	1.05	0.069
Unknown	1.29	0.9	1.84	0.169				

* **ER** Estrogen Receptor, **PR** Progesterone receptor, **HR** Hormone Receptor, **TNBC** Triple Negative Breast Cancer; CI: confidence interval, **CI** confidence interval, **IDC** invasive ductal carcinoma,** **PR** Prevalence ratio

Supplementary Table S5. Association of metabolic syndrome with tumor subtypes by Hispanic subgroups.

**ER***	**Mexican Hispanics**				**Non-Mexican Hispanics**			
	**PR****	**95%CI**		**p-value**	**PR****	**95%CI**		**p-value**
Metabolic syndrome	1.12	1.02	1.24	0.023	1.13	0.91	1.40	0.28
Postmenopausal	1.05	0.94	1.17	0.385	0.77	0.66	0.90	0.001
Lobular	1.45	1.34	1.57	0	1.08	0.85	1.37	0.536
Mixed	1.10	0.76	1.61	0.608	1.11	0.82	1.50	0.489
Unknown	1.09	0.90	1.32	0.376	1.00	0.72	1.39	0.999
Advanced stage	0.85	0.74	0.97	0.016	0.93	0.76	1.13	0.439
Unknown	1.06	0.96	1.18	0.246	0.86	0.70	1.06	0.159
Lumpectomy	1.15	0.93	1.44	0.203	0.99	0.75	1.31	0.948
Mastectomy	1.04	0.84	1.31	0.7	1.10	0.85	1.41	0.479
Unknown	1.12	0.48	2.64	0.793	0.89	0.51	1.56	0.694
**PR***	**Mexican Hispanics**				**Non-Mexican Hispanics**			
Metabolic syndrome	1.21	1.06	1.37	0.004	1.21	0.92	1.60	0.174
Postmenopausal	0.94	0.83	1.07	0.378	0.69	0.55	0.86	0.001
Lobular	1.50	1.31	1.73	0	0.99	0.69	1.41	0.957
Mixed	1.31	0.90	1.90	0.156	1.03	0.66	1.62	0.89
Unknown	1.13	0.89	1.43	0.317	0.77	0.41	1.46	0.426
Advanced stage	0.85	0.73	0.99	0.042	0.96	0.72	1.27	0.776
Unknown	1.02	0.89	1.17	0.747	0.92	0.70	1.20	0.538
Lumpectomy	1.12	0.86	1.46	0.389	1.01	0.67	1.54	0.952
Mastectomy	1.03	0.79	1.34	0.823	1.21	0.83	1.76	0.312
Unknown	1.37	0.57	3.29	0.482	1.12	0.61	2.08	0.711
**HR***	**Mexican Hispanics**				**Non-Mexican Hispanics**			
Metabolic syndrome	1.13	1.02	1.25	0.02	1.13	0.91	1.40	0.28
Postmenopausal	1.03	0.93	1.15	0.535	0.77	0.66	0.90	0.001
Lobular	1.38	1.25	1.52	0	1.08	0.85	1.37	0.536
Mixed	0.99	0.63	1.54	0.951	1.11	0.82	1.50	0.489
Unknown	0.97	0.78	1.19	0.745	1.00	0.72	1.39	0.999
Advanced stage	0.86	0.76	0.98	0.028	0.93	0.76	1.13	0.439
Unknown	1.02	0.92	1.14	0.668	0.86	0.70	1.06	0.159
Lumpectomy	1.10	0.89	1.36	0.382	0.99	0.75	1.31	0.948
Mastectomy	1.00	0.81	1.23	0.977	1.10	0.85	1.41	0.479
Unknown	0.84	0.31	2.25	0.722	0.89	0.51	1.56	0.694
**TNBC***	**Mexican Hispanics**				**Non-Mexican Hispanics**			
Metabolic syndrome	0.93	0.64	1.35	0.693	0.79	0.33	1.94	0.611
Postmenopausal	0.75	0.56	1.00	0.054	2.17	1.07	4.39	0.031
Lobular	0.09	0.01	0.65	0.017	0.79	0.33	1.89	0.596
Mixed	0.75	0.13	4.47	0.756	0.95	0.24	3.79	0.946
Unknown	0.81	0.44	1.52	0.519	1.05	0.25	4.46	0.947

Advanced stage	1.01	0.73	1.39	0.975	1.39	0.62	3.13	0.42
Unknown	0.91	0.62	1.34	0.642	1.95	0.99	3.86	0.054
Lumpectomy	0.54	0.34	0.86	0.009	1.09	0.53	2.21	0.819
Mastectomy	0.65	0.42	1.01	0.055	0.53	0.24	1.19	0.125
Unknown	1.47	0.36	5.92	0.591	0.65	0.12	3.47	0.61

ER Estrogen Receptor, **PR** Progesterone receptor, **HR** Hormone Receptor, **TNBC** Triple-Negative Breast Cancer, **CI** confidence interval, **IDC** invasive ductal carcinoma, ****PR** Prevalence ratio
